# The Association Between Diabetes Mellitus and the Risk of Latent Tuberculosis Infection: A Systematic Review and Meta-Analysis

**DOI:** 10.3389/fmed.2022.899821

**Published:** 2022-04-25

**Authors:** Qiao Liu, Wenxin Yan, Runqing Liu, Ershu Bo, Jue Liu, Min Liu

**Affiliations:** ^1^Department of Epidemiology and Biostatistics, School of Public Health, Peking University, Beijing, China; ^2^School of Health Humanities, Peking University, Beijing, China; ^3^School of Basic Medicine, Peking University, Beijing, China

**Keywords:** tuberculosis infection, diabetes mellitus, meta-analysis, prediabetes, systematic review

## Abstract

**Background:**

The estimated global latent tuberculosis infection (LTBI) burden indicates a large reservoir of population at risk of developing active tuberculosis (TB). Previous studies suggested diabetes mellitus (DM) might associate with LTBI, though still controversial. We aimed to systematically assess the association between DM and LTBI.

**Methods:**

We searched PubMed, Embase, Cochrane Library and Web of Science. Observational studies reporting the number of LTBI and non-LTBI individuals with and without DM were included. Random-effects or fixed-effects models were used to estimate the pooled effect by risk ratios (RRs) and odds ratios (ORs) and its 95% confidence interval (CI), using the original number of participants involved.

**Results:**

20 studies involving 4,055,082 participants were included. The pooled effect showed a significant association between DM and LTBI (for cohort studies, *RR* = 1.62, 95% CI: 1.02–2.56; for cross-sectional studies, *OR* = 1.55, 95% CI: 1.30–1.84). The pooled OR was high in studies with healthcare workers (5.27, 95% CI: 1.52–8.20), refugees (2.88, 95% CI: 1.93–4.29), sample size of 1,000–5,000 (1.99, 95% CI: 1.49–2.66), and male participants accounted for less than 40% (2.28, 95% CI: 1.28–4.06). Prediabetes also associated with LTBI (*OR* = 1.36, 95% CI: 1.01–1.84).

**Conclusion:**

The risk of LTBI was found to be a 60% increase in DM patients, compared with non-DM patients. LTBI screening among DM patients could be of vital importance. More studies are needed to explore appropriate strategies for targeted LTBI screening among DM patients.

## Introduction

Tuberculosis (TB) is a major infectious disease and one of the top ten causes of death worldwide, which has caused 1.4 million deaths in 2020 (1). Latent tuberculosis infection (LTBI) is a state of persistent immune response to stimulation by *M. tuberculosis* antigens with no evidence of clinically manifest Tuberculosis disease (2). Several studies showed the global prevalence of LTBI was over 20%, and the proportion came to over 30% in areas with high TB incidence (3–5). Active TB disease is the leading cause of death from a single infectious agent (ranking above HIV/AIDS), despite being curable and preventable (1). WHO estimates that 5–15% of LTBI individuals will fall ill with active TB disease (5). Moreover, the estimated global LTBI burden clearly indicates a large reservoir of population at risk of developing active TB. WHO aimed to end the global TB epidemic, but most countries failed to reach the 2020 milestones of the End TB Strategy, which required a 20% reduction of TB incidence rate between 2015 and 2020 (1, 6). For high-risk populations, TB preventive treatment (TPT) could reduce the risk of developing active TB disease, which emphasized the importance of identifying high-risk populations for LTBI testing and TPT (2).

Diabetes mellitus (DM) is a chronic and metabolic disease characterized by elevated levels of blood glucose (or blood sugar), which can impair the immunity of individuals and increase the susceptibility to various infectious diseases (7, 8). The global prevalence of DM was rising and it was the eighth leading cause of global disability-adjusted life-years in 2019 (9). In 2015, an estimated 8.8% or 415 million people were living with DM worldwide, nearly double the 4.6% (151 million) estimated in 2000, and this number was expected to increase to 10.4% (642 million) by 2040 (10). The association between DM and the risk of active TB has been well established (11). Previous studies had shown that DM could triple the risk of developing TB disease, leading to adverse TB treatment outcomes such as prolongation of culture conversion, treatment failure, relapse, and death (12–14).

However, people with DM are not recommended to get systematic LTBI testing and TPT, unless they also belong to other risk groups (2). Evidence on the association between DM and the risk of LTBI was still limited. Previous meta-analyses had shown that DM was associated with the risk of TB (15, 16), yet the association between DM and LTBI was still unclear. Recently, global attention to the association between DM and the risk of LTBI has increased, and there were more observational studies published in recent years, especially for cohort studies (17–25). We aimed to systematically assess the association between DM and the risk of LTBI, as well as prediabetes (pre-DM), to provide the latest evidence on early screening of LTBI and prevention of its progress to active TB among DM patients.

## Materials and Methods

### Data Sources and Search Strategy

Our methods have been described in detail in our published protocol (26) [PROSPERO (Prospective register of systematic reviews) registration, CRD42021244647]. We searched for eligible studies published by 31 December 2021, from four databases including PubMed, Embase, Web of Science and Cochrane Library by the following search terms: (“tuberculin test” or “interferon-gamma release tests” or “latent tuberculosis” or “tuberculin skin test” or “interferon gamma release assay”) AND (“diabetes mellitus” or “diabetes”) OR “latent tuberculosis” (full search strategies were shown in Supplementary Table 1). We used EndNote X9.0 software to manage records, screen, and exclude duplicates. This study was strictly performed according to the Meta-analysis of Observational Studies in Epidemiology (MOOSE) (27).

### Inclusion and Exclusion Criteria

We included articles that met the following criteria: (1) cohort studies and cross-sectional studies, (2) studies reporting the number of LTBI patients with and without DM, and non-LTBI people with and without DM. Articles were excluded if met any following criteria: (1) irrelevant to the subject of the meta-analysis, (2) studies with insufficient data to calculate the odds ratio or assess the pooled effect of diabetes mellitus on risk of latent tuberculosis infection, (3) duplicated or overlapped articles, (4) studies in lack of the definition of DM or LTBI patients, and (5) if articles were not written in English or Chinese.

Latent tuberculosis infection: LTBI, is a state of persistent immune response to stimulation by *M. tuberculosis* antigens with no evidence of clinically manifest Tuberculosis (TB) disease (2).

Diabetes mellitus/Prediabetes: DM, is a chronic and metabolic disease characterized by elevated levels of blood glucose (or blood sugar) (8). With prediabetes, blood sugar levels are higher than normal, but not high enough yet to be diagnosed as type 2 DM (28).

Studies were identified by four investigators (LQ, YWX, LRQ, and BES) independently following the criteria above, and each abstract and full text article was reviewed in duplicate by at least two reviewers. Discrepancies were reconciled by a fifth investigator (LJ).

### Quality Assessment

We evaluated the risk of bias using the Newcastle-Ottawa quality assessment scale for cohort studies and case-control studies (29). Cohort studies and case-control studies were classified as having low (≥7 stars), moderate (5–6 stars), and high risk of bias (≤4 stars) with an overall quality score of 9 stars (30, 31). For cross-sectional studies, we assigned each item of the AHRQ checklist a score of 1 (answered “yes”) or 0 (answered “no” or “unclear”), and summarized scores across items to generate an overall quality score that ranged from 0 to 11. Low, moderate, and high risk of bias were identified as having a score of 8–11, 4–7, and 0–3, respectively. Two investigators (LQ and YWX) assessed study quality independently, with disagreements resolved by a third investigator (LJ).

### Data Extraction

The primary outcome was the impact of DM on LTBI in different people groups. Data were extracted using a predesigned form that had been piloted. The following data were extracted independently by four investigators (LQ, YWX, LRQ, and BES) from the selected studies: (1) basic information of the studies including first author, publication year and research type; (2) characteristics of the study population including sample sizes and locations; (3) primary outcomes: the number of people with or without DM in the total population and by laboratory diagnosis results of LTBI; and (4) diagnostic methods of LTBI: tuberculin skin test (TST), QuantiFERON-TB Gold In-Tube test (QFT-GIT), and interferon gamma release assays (IGRAs). The data in each article were extracted independently by at least two investigators, and discrepancies were reconciled by a fifth investigator (LJ).

### Data Synthesis and Statistical Analysis

We performed a meta-analysis to summarize data from cohort and cross-section studies and assessed the overall effect of DM on LTBI, using the original number of participants involved and adjusted OR across the studies. Fixed-effects or random-effects models were used to obtain the pooled effect across studies, according to the heterogeneity between estimates. Fixed-effects models were used if *I*^2^ ≤ 50%, *P* > 0.10, which represents insignificant heterogeneity; while random-effects models used if *I*^2^ ≥ 50%, *P* < 0.10, representing significant heterogeneity.

The pooled effect of DM on LTBI was described by forests plots, quantified by OR/RR (Unable to calculate the incidence rate, cross-sectional studies were pooled by OR, the ratio between the odds of exposure in diseased and in non-diseased individuals; and cohort studies are pooled by RR, the ratio between the rates of disease in exposed and non-exposed individuals), and the corresponding 95% confidence intervals (CI). A value of *P* < 0.05 was considered significant. If substantial heterogeneity was observed, we conducted subgroup analyses to investigate the possible sources of heterogeneity, by using population group, study location, sample size, proportion of male, and risk of bias as grouping variables. We used the *Q*-test to conduct subgroup comparisons and variables were considered significant between subgroups if the subgroup difference *p*-value was less than 0.05. Sensitivity analyses were performed by omitting one study at a time, trying to discover articles that have notable impact and examine the stability of the overall effect. Publication bias was assessed by funnel plot and Egger’s regression test. Additionally, we extracted information about pre-DM patients and also performed a meta-analysis to investigate if there was any association between pre-DM and the risk of LTBI. We analyzed data using Stata version 16.0.

## Results

### Study Selection and Study Characteristics

**FIGURE 1:**
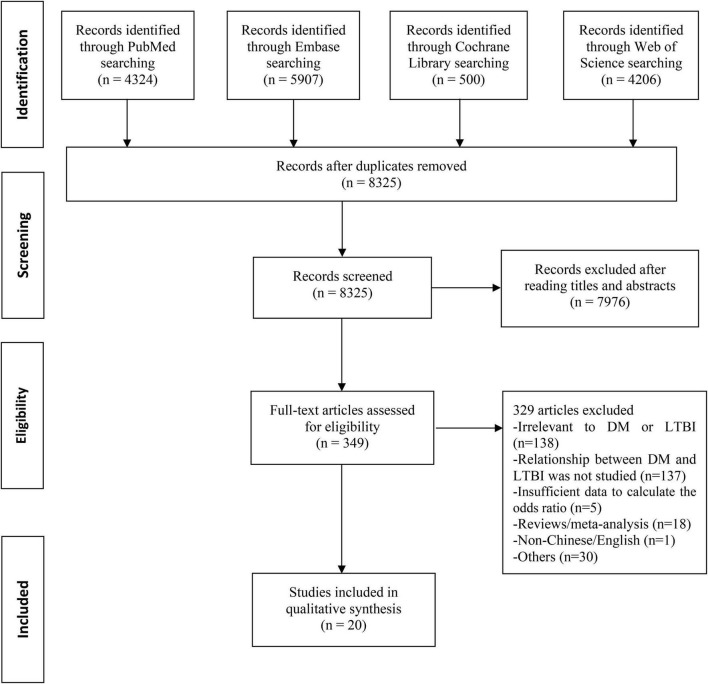
Flowchart of the study selection. DM, diabetes mellitus; LTBI, latent tuberculosis infection.

A total of 14,937 records were searched from the four databases by terms mentioned above. 6,612 duplicates were excluded. After reading titles and abstracts, we excluded 7,976 reviews, conference papers, animal experiments, case reports, and other studies irrelevant to the subject. Among the 349 articles under full-text review, 329 articles were excluded for lacking targeted data or meeting any one of the exclusion criteria (Figure 1). Ultimately, this final meta-analysis comprised 20 eligible studies (Table 1 and Supplementary Table 2) (17–25, 32–42), including three cohort studies and 17 cross-sectional studies. The studies involved a total sample size of 4,055,082 (243,253 patients with DM and 3,811,829 people without DM) from nine countries. And 15 studies were assessed as having low risk of bias, with five studies having moderate risk of bias (Supplementary Table 3).

**TABLE 1:** Characteristics of the studies included for meta-analysis.

References	Location	Design	Sample size	Population	Proportion of male	DM _LTBI	DM _nonLTBI	NonDM _LTBI	NonDM _nonLTBI	Risk of bias
Arnedo-Pena et al. (32)	Spain	Cohort study	198	Contacts	50.6%	1	3	17	177	Low
Salindri et al. (38)	United States	Cross-sectional study	132	Community residents	44.7%	9	89	5	29	Moderate
Barron et al. (18)	United States	Cross-sectional study	4958	Community residents	47.8%	65	500	152	3145	Low
Bennett et al. (33)	United States	Cross-sectional study	4187	Refugees	50.5%	76	100	747	3264	Low
Chan-Yeung et al. (33)	HK, China	Cross-sectional study	3605	Residents in old age homes	27.5%	423	1245	383	1554	Moderate
El-Sokkary et al. (35)	Egypt	Cross-sectional study	132	Health care workers	22.7%	20	10	18	84	Moderate
Hensel et al. (36)	United States	Cross-sectional study	681	Refugees	55.0%	23	31	104	302	Low
Jackson et al. (19)	United Kingdom	Cross-sectional study	9157	Community residents	50.0%	238	518	2296	6105	Low
Khawcharoenporn et al. (37)	Thailand	Cohort study	150	HIV-infected patients	52.7%	2	4	34	110	Low
Kubiak et al. (23)	India	Cross-sectional study	1113	Household contacts	35.2%	44	25	561	483	Low
Lin et al. (20)	Taiwan, China	Cross-sectional study	3401	community residents	46.4%	623	2325	44	409	Low
Martinez et al. (22)	United States	Cross-sectional study	4215	Population	49.2%	59	717	82	1916	Low
Nanth et al. (21)	Malaysia	Cross-sectional study	763	Non-communicable disease patients from clinic	49.1%	115	289	105	254	Low
Shivakumar et al. (17)	India	Cross-sectional study	639	Household contacts	43.6%	47	16	321	81	Low
Shu et al. (39)	Taiwan, China	Cross-sectional study	427	Adult patients receiving long-term dialysis	53.0%	24	84	67	252	Moderate
Stockbridge et al. (25)	United States	Cross-sectional study	3997986	Household contacts	49.4%	11225	222,637	161857	3602267	Low
Suwanpimolkul et al. (40)	United States	Cross-sectional study	23018	Individuals seeking medical attention at the Tuberculosis Clinic	Unknown	1158	332	16698	4830	Low
Ting et al. (41)	Taiwan, China	Cross-sectional study	1018	In-patients and out- patients	52.5%	48	99	248	623	Low
Wang et al. (42)	Taiwan, China	Cohort study	583	Household contacts	33.3%	8	9	168	398	Low
Yeon et al. (24)	South Korea	Cross-sectional study	1655	Healthcare workers	25.6%	4	8	263	1376	Moderate

*DM, diabetes mellitus; LTBI, latent tuberculosis infection.*

### Effects of Diabetes Mellitus on Latent Tuberculosis Infection

**FIGURE 2:**
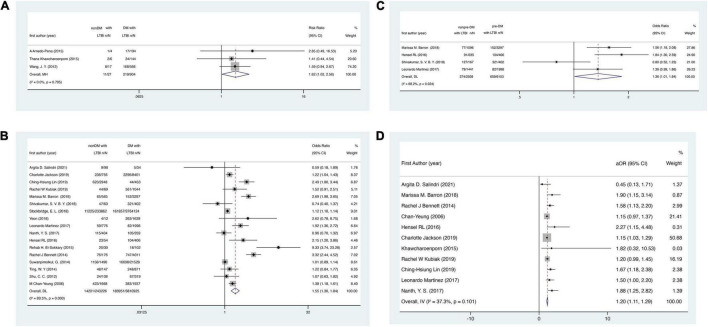
Forest plots for the association between DM/pre-DM and the risk of LTBI. **(A)** Association between DM and the risk of LTBI in cohort studies. **(B)** Association between DM and the risk of LTBI in cross-sectional studies. **(C)** Association between pre-DM and the risk of LTBI. **(D)** Association between DM and the risk of LTBI with adjusted ORs. DM, diabetes mellitus; LTBI, latent tuberculosis infection.

In the cohort studies, DM was associated with 62% higher risk of LTBI, with the pooled risk ratio (RR) 1.62 (95% CI: 1.02–2.56, Figure 2A). In the cross-sectional studies, DM was also associated with the risk of LTBI (pooled *OR* = 1.55, 95% CI: 1.30–1.84, Figure 2B). A significant association between pre-DM and the risk of LTBI was also observed (pooled *OR* = 1.36, 95% CI: 1.01–1.84, Figure 2C) among four studies (17, 18, 22, 36) in which we could extract related information. Additionally, when pooling data with adjusted ORs, the association was also observed (pooled *OR* = 1.20, 95% CI, 1.11–1.29, Figure 2D).

### Subgroup Analysis of Cross-Sectional Studies

Given the substantial heterogeneity among the studies, we did subgroup analyses by population group, study location, sample size, proportion of male, and study quality for cross-sectional studies (Table 2). In the subgroup analysis, the *P*-values for subgroup differences of population group, study location, sample size, and proportion of male were all lower than 0.01. And the pooled effect was significantly higher in studies with healthcare workers (HCWs) (*OR* = 5.27, 95% CI: 1.52–18.20), carried out in Africa (*OR* = 9.33, 95% CI: 3.74–23.28), with a sample size of between 1,000 and 5,000 (*OR* = 1.99, 95% CI: 1.49–2.66), with male accounted for less than 40% (*OR* = 2.36, 95% CI: 1.19–4.66). There were no significant differences in studies classified by the risk of bias (low: *OR* = 1.53, 95% CI: 1.25–1.86; moderate: *OR* = 1.78, 95% CI: 0.93–3.42; p = 0.658). Population group, study location, sample size, and proportion of male might be the potential source of heterogeneity in the association between DM and the risk of LTBI.

**TABLE 2:** Pooled effect for cohort and cross-sectional studies, and subgroup analysis for cross-sectional studies by population group, study location, sample size, proportion of male, study quality.

	No. studies	Odds ratio (95% CI)	*I* ^2^	*P*-value for heterogeneity	*P*-value for subgroup differences	Weight (%)
**Study design**						
Cohort studies	3	1.62 (1.02, 2.56)*	<0.1%	0.795		.
Cross-sectional studies	17	1.55 (1.30, 1.84)	89.5%	<0.001		.
**Population group**					< 0.001	
Community residents	5	1.77 (1.16, 2.70)	87.9%	<0.001		30.70
Contacts	3	1.12 (0.88, 1.43)	35.2%	0.214		18.33
Refugees	2	2.88 (1.93, 4.29)	39.4%	0.199		11.48
Elderly/patients with other chronic diseases	5	1.13 (0.95, 1.35)	61.7%	0.034		35.24
Health care worker	2	5.27 (1.52, 18.20)	63.1%	0.100		4.25
**Study location**					<0.001	
Europe	1	1.22 (1.04, 1.43)				8.37
Asia	8	1.33 (1.03, 1.72)	71.1%	0.001		44.49
North America	7	1.69 (1.25, 2.29)	93.8%	<0.001		44.56
Africa	1	9.33 (3.74, 23.28)				2.57
**Sample size**					0.001	
100–1,000	6	1.41 (0.78, 2.55)	82.8%	<0.001		24.89
1,000–5,000	8	1.99 (1.49, 2.66)	83.3%	<0.001		49.11
>5,000	3	1.11 (1.03, 1.20)	47.2%	0.151		26.00
**Proportion of male**					0.010	
<40%	4	2.36 (1.19, 4.66)	82.6%	0.001		17.76
40–50%	7	1.42 (0.99, 2.05)	91.1%	<0.001		42.51
≥ 50%	5	1.63 (1.03, 2.57)	88.8%	<0.001		31.12
Unknown	1	1.01 (0.89, 1.14)				8.61
**Risk of bias**					0.658	
Low	12	1.53 (1.25, 1.86)	91.1%	<0.001		80.67
Moderate	5	1.78 (0.93, 3.42)	80.7%	<0.001		19.33

*This effect is Risk Ratio.

### Sensitivity Analysis and Publication Bias

**FIGURE 3:**
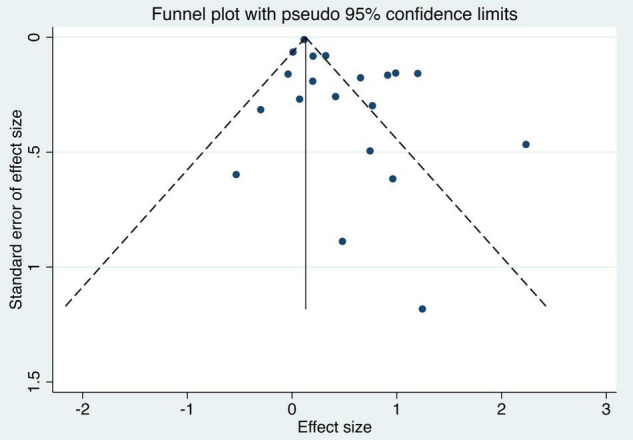
Funnel plot of the studies based on the association between DM and the risk of LTBI. DM, diabetes mellitus; LTBI, latent tuberculosis infection.

In the sensitivity analyses, the pooled results were consistent and all the results showed that DM has an effect on LTBI when omitting one study at a time, which demonstrated that the pooled estimation was stable. Funnel plot was shown in Figure 3. Egger’s regression test (*t* = 2.87, *p* = 0.01) indicated that publication bias might exist.

## Discussion

In this meta-analysis, a total of 20 studies were included. The pooled RR for the 3 cohort studies was 1.62 (95% CI: 1.02–2.56), and the pooled OR for the 17 cross-sectional studies was 1.55 (95% CI: 1.30–1.84), both revealing a significant increased risk of LTBI among DM patients. A previous meta-analysis conducted by Lee and colleagues identified 13 cross-sectional studies and 1 cohort study (15). They found a small but statistically significant pooled OR (1.18, 95% CI: 1.06–1.30) for the cross-sectional studies, which was consistent with our findings (15). However, they found an increased but non-significant risk of LTBI among DM patients (*RR* = 4.40, 95% CI, 0.50–38.55) for a cohort study, which was contradictory to our study (15). Moreover, the pooled OR for cross-sectional studies and pooled RR for cohort studies in our study were similar, indicating the stability and reliability of our results. Notably, 2 of the 3 cohort studies were conducted among contacts of active TB patients (32, 42), while the left 1 was in HIV-infected patients (37). Both people living with HIV and adult contacts of TB disease had an increased risk of progression from LTBI to active TB (43). Therefore, our findings indicated that more attention should be paid to these high-risk groups (such as HIV-infected patients) when they also had DM.

Due to hyperglycemia, insulin resistance, macrophage and lymphocyte function, and abnormal homeostatic levels of cytokines such as type 1 and type 17 cytokines, DM patients have enhanced susceptibility to TB (44–47). In addition, in LTBI patients, DM could reduce protective CD4^+^ and CD8^+^ T-cell responses (48, 49). These T-cells could produce IFN-γ, TNF-α, and IL-2, which were proved in previous studies to have critical functions during *M. tuberculosis* infection (50, 51). In DM patients, lower frequencies of myeloid and plasmacytoid dendritic cells (DC), and classical and intermediate monocytes, as well as significantly higher frequencies of non-classical monocytes were also observed in another study, compared with individuals without DM, suggesting that coincident DM could profoundly alter the frequencies of monocyte and DC subsets in LTBI (52). In addition, we must consider the confounding factor of age, on the one hand, type 2 DM is most frequently diagnosed in the elderly, whilst on the other hand, immune senescence appears to reduce their ability to generate sufficient immune T-lymphocytes to prevent the recurrence of tuberculosis (53), resulting in susceptibility to LTBI.

In the subgroup by population group, the pooled effect of DM was significantly higher in studies with HCWs, refugees, and community residents, compared to studies with TB contacts, elderly or patients with other chronic diseases. The *p*-value for the subgroup difference was less than 0.001, indicating that the population group might be the potential source of heterogeneity for the association between DM and the risk of LTBI. Previous studies of Baussano et al. (54) and Nasreen et al. (55) had shown that HCWs were at increased risk of LTBI from occupational exposure to *M. tuberculosis*, which explained why HCWs with DM had greater risk of LTBI than other population groups. In group refugees, two studies were conducted in United States, including refugees consulted in DeKalb County Board of Health Refugee Clinic (36) and resettled in San Diego (33). Among the refugee community, overcrowding was associated with increased transmission of pulmonary disease, and other factors contributing to increased risk of increased transmission and morbidity included malnutrition, disrupted health services, lack of TB medications, and comorbidities such as HIV, Hepatitis, vitamin D deficiency, and DM, along with lack of water, sanitation, social services, and education (56). Mild malnutrition could even increase the risk of tuberculosis progression and case fatality. Ahmad M’s (56) retrospective study suggested refugees, especially consolidated with DM, had greater risk of LTBI than community residents, which explained the heterogeneity in the association between DM and the risk of LTBI.

In the subgroup analysis by study location, the pooled effect was significantly higher in studies carried out in Africa and North America, compared with studies carried out in Asia and Europe. HCW was the main population in the Africa group (35), which was the only study and led a high odds ratio of this group. Population of group North America consisted of refugees (33, 36), community residents (18, 22), elderly or patients with other chronic diseases (40) and household contacts (25). These four populations led to high heterogeneity in the whole group. Among them, strong effects of refugees (33, 36) and community residents (18, 22) were the main reasons resulting in the high risk of LTBI in the whole group. The heterogeneity of group North America was even greater than the overall heterogeneity, which suggested that these studies might be the potential source of heterogeneity in the association between DM and the risk of LTBI.

In the subgroup by sample size, the pooled effect was significantly higher in studies with a sample size between 1,000 and 5,000, compared to studies with a sample size over 5000, indicating that the sample size of studies may have impact on the conclusion. The source of heterogeneity might be explained by Zhang et al. (57) who suggested that trials with smaller sample sizes were more likely to report larger beneficial effects than large trials. In the subgroup by proportion of male, the pooled effect significantly was higher in studies with a proportion of male less than 40%, compared to studies with a proportion of male between 40 and 50% and no less than 50%, indicating that gender might have impact on the effect of DM on risk of LTBI. Currently, study on the gender difference of risk of LTBI was scarce. But accessing the database (58), we found LTBI prevalence was generally higher in males than in females, suggesting there were more risk factors for males to develop the disease, so we speculated the high LTBI prevalence in male controls reduced the effect of DM, explaining why studies with males less than 40% had a significantly higher pooled effect.

Our results also showed that pre-DM had a small but statistically significant effect of associating with the risk of LTBI (pooled *OR* = 1.36, 95% CI: 1.01–1.84). Pre-DM, or intermediate hyperglycemia, is a high-risk state of DM that is defined by glucose values higher than normal, but lower than DM threshold (59). The prevalence of pre-DM was increasing globally, and it was estimated that over 470 million people will have pre-DM by 2030 (60). Previous study had shown that pre-DM was associated with alterations of the immune response in LTBI associated with compromised CD4^+^ and CD8^+^ T-cell function (61). However, like people with DM, there was a paucity of data in pre-DM patients from clinical trials on the relative benefits and harm of systematic testing and tuberculosis preventive treatment (TPT), and whether to provide testing and treatment among people with DM and pre-DM depended on a case-by-case basis (2). More studies and evidence on systematic TPT services among people with DM and pre-DM are needed to understand the potential impact of an LTBI screening program for populations with DM and pre-DM. Additionally, previous studies had demonstrated that pre-DM was also associated with risk of cardiovascular disease and heart failure (62–65). This available evidence emphasized the impact of pre-DM on people’s health and that we should pay more attention to the management of pre-DM.

DM also tripled the risk of developing active TB disease, leading to adverse TB treatment outcomes such as prolongation of culture conversion, treatment failure, relapse, and death (12–14). This fact emphasized the importance of LTBI screening in populations with DM. Currently, countries with low TB burden also attached importance to the TPT of LTBI (43). However, DM patients were not recommended by WHO to get systematic testing and TPT (2). In recent years, the increasing number of studies accumulated evidence of the association between DM and the risk of LTBI. In our study, the pooled effects of cross-sectional and cohort studies were highly consistent, both revealing that the risk of LTBI was found to be a 60% increase in DM patients, compared with individuals without DM. Our findings could be considered when assessing the potential impact of LTBI screening and TPT among populations with DM.

Our study has limitations. First, for the cross-sectional studies included, publication bias exists. Second, tuberculin skin test and interferon gamma release assays (or both) were used to identify the status of LTBI in all studies, since there was no gold standard test for direct identification of LTBI in humans (2). However, there was no strong evidence that one test should be preferred over the other in terms of predicting progression from LTBI to TB disease (2). Third, most studies verified the status of DM by self-report or medical record. DM was often underdiagnosed and undertreated, which would likely result in non-differential misclassification of DM, making the DM-LTBI association underestimated (66). Fourth, most studies were conducted in specific population, thus our finding might not be generalizable to general population. Fifth, the heterogeneity among studies was high, which might be related to different study population, location, sample size, and proportion of male.

## Conclusion

The risk of LTBI was found to be a 60% increase in DM patients, compared with individuals without DM. Pre-DM was also associated with increased risk of LTBI. Our findings highlight the importance of LTBI screening among DM patients. More studies are needed to explore appropriate strategies and tools for targeted LTBI screening among DM patients, thereby decreasing global burden of TB disease and improving the prognosis of DM.

## Data Availability Statement

The original contributions presented in the study are included in the article/Supplementary Material, further inquiries can be directed to the corresponding author/s.

## Author Contributions

JL and ML conceived and designed the study. QL and RL carried out the literature searches. QL, WY, RL, and EB extracted the data. QL, WY, and JL assessed the study quality. QL and WY performed the statistical analysis and wrote the manuscript. JL, ML, QL, and WY revised the manuscript. All authors contributed to the article and approved the submitted version.

## Conflict of Interest

The authors declare that the research was conducted in the absence of any commercial or financial relationships that could be construed as a potential conflict of interest.

## Publisher’s Note

All claims expressed in this article are solely those of the authors and do not necessarily represent those of their affiliated organizations, or those of the publisher, the editors and the reviewers. Any product that may be evaluated in this article, or claim that may be made by its manufacturer, is not guaranteed or endorsed by the publisher.

## References

[1] World Health Organization. *Global Tuberculosis Report 2020.* Geneva: World Health Organization (2020).

[2] World Health Organization. *WHO Operational Handbook on Tuberculosis.* Geneva: World Health Organization (2021).

[3] Houben RM Dodd PJ. The global burden of latent tuberculosis infection: a re-estimation using mathematical modelling. *PLoS Med.* (2016) 13:e1002152. 10.1371/journal.pmed.1002152 27780211 PMC5079585

[4] Cohen A Mathiasen VD Schön T Wejse C. The global prevalence of latent tuberculosis: a systematic review and meta-analysis. *Eur Respir J.* (2019) 54:1900655. 10.1183/13993003.00655-2019 31221810

[5] World Health Organization. *Tuberculosis: Overview.* Geneva: World Health Organization (2021).

[6] World Health Organization. *The WHO End TB Strategy.* Geneva: World Health Organization (2021).

[7] Martinez N Kornfeld H. Diabetes and immunity to tuberculosis. *Eur J Immunol.* (2014) 44:617–26. 10.1002/eji.201344301 24448841 PMC4213860

[8] World Health Organization. *Diabetes: Overview.* Geneva: World Health Organization (2021).

[9] Tisdell CA. Economic, social and political issues raised by the COVID-19 pandemic. *Econ Anal Policy.* (2020) 68:17–28. 10.1016/j.eap.2020.08.002 32843816 PMC7440080

[10] Koye DN Magliano DJ Nelson RG Pavkov ME. The global epidemiology of diabetes and kidney disease. *Adv Chronic Kidney Dis.* (2018) 25:121–32. 10.1053/j.ackd.2017.10.011 29580576 PMC11000253

[11] Hayashi S Chandramohan D. Risk of active tuberculosis among people with diabetes mellitus: systematic review and meta-analysis. *Trop Med Int Health.* (2018) 23:1058–70. 10.1111/tmi.13133 30062731

[12] Salindri AD Kipiani M Kempker RR Gandhi NR Darchia L Tukvadze N Diabetes reduces the rate of sputum culture conversion in patients with newly diagnosed multidrug-resistant tuberculosis. *Open Forum Infect Dis.* (2016) 3:ofw126. 10.1093/ofid/ofw126 27419188 PMC4942763

[13] Shewade HD Jeyashree K Mahajan P Shah AN Kirubakaran R Rao R Effect of glycemic control and type of diabetes treatment on unsuccessful TB treatment outcomes among people with TB-diabetes: a systematic review. *PLoS One.* (2017) 12:e0186697. 10.1371/journal.pone.0186697 29059214 PMC5653348

[14] Baker MA Harries AD Jeon CY Hart JE Kapur A Lönnroth K The impact of diabetes on tuberculosis treatment outcomes: a systematic review. *BMC Med.* (2011) 9:81. 10.1186/1741-7015-9-81 21722362 PMC3155828

[15] Lee MR Huang YP Kuo YT Kuo YT Luo CH Shih YJ Diabetes mellitus and latent tuberculosis infection: a systematic review and meta analysis. *Clin Infect Dis.* (2017) 64:719–27. 10.1093/cid/ciw836 27986673 PMC5399944

[16] Foe-Essomba JR Kenmoe S Tchatchouang S Ebogo-Belobo JT Mbaga DS Bowo-Ngandji A Diabetes mellitus and tuberculosis, a systematic review and meta-analysis with sensitivity analysis for studies comparable for confounders. *PLoS One.* (2021) 16:e0261246. 10.1371/journal.pone.0261246 34890419 PMC8664214

[17] Shivakumar SVBY Chandrasekaran P Kumar AMV Paradkar M Dhanasekaran K Suryavarshini N Diabetes and pre-diabetes among household contacts of tuberculosis patients in India: is it time to screen them all? *Int J Tuberc Lung Dis.* (2018) 22:686–94. 10.5588/ijtld.17.0598 29862955

[18] Barron MM Shaw KM Bullard KM Ali MK Magee MJ. Diabetes is associated with increased prevalence of latent tuberculosis infection: findings from the national health and nutrition examination survey, 2011–2012. *Diabetes Res Clin Pract.* (2018) 139:366–79. 10.1016/j.diabres.2018.03.022 29574108

[19] Jackson C Southern J Lalvani A Drobniewski F Griffiths CJ Lipman M Diabetes mellitus and latent tuberculosis infection: baseline analysis of a large UK cohort. *Thorax.* (2019) 74:91–4. 10.1136/thoraxjnl-2017-211124 29764958

[20] Lin CH Kuo SC Hsieh MC Ho SY Su IJ Lin SH Effect of diabetes mellitus on risk of latent TB infection in a high TB incidence area: a community-based study in Taiwan. *BMJ Open.* (2019) 9:e029948. 10.1136/bmjopen-2019-029948 31662365 PMC6830704

[21] Nanth YS Puri A Ali SZM Suppiah P Che Ali SA Ramasamy B Epidemiology of latent tuberculosis infection among patients with and without diabetes mellitus. *Fam Pract.* (2017) 34:532–8. 10.1093/fampra/cmx017 28369346

[22] Martinez L Zhu L Castellanos ME Liu Q Chen C Hallowell BD Glycemic control and the prevalence of tuberculosis infection: a population-based observational study. *Clin Infect Dis.* (2017) 65:2060–8. 10.1093/cid/cix632 29059298 PMC5848314

[23] Kubiak RW Sarkar S Horsburgh CR Roy G Kratz M Reshma A Interaction of nutritional status and diabetes on active and latent tuberculosis: a cross-sectional analysis. *BMC Infect Dis.* (2019) 19:627. 10.1186/s12879-019-4244-4 31311495 PMC6636094

[24] Yeon JH Seong H Hur H Park Y Kim YA Park YS Prevalence and risk factors of latent tuberculosis among Korean healthcare workers using wholeblood interferon-gamma release assay. *Sci Rep.* (2018) 8:10113. 10.1038/s41598-018-28430-w 29973678 PMC6031657

[25] Stockbridge EL Miller TL Carlson EK Ho C. Private sector tuberculosis prevention in the US: characteristics associated with interferon-gamma release assay or tuberculin skin testing. *PLoS One.* (2018) 13:e0193432. 10.1371/journal.pone.0193432 29590130 PMC5873986

[26] Liu M Liu J Liu Q Yan W. *The Association Between Diabetes Mellitus and the Risk of Latent Tuberculosis Infection: A Systematic Review and Meta-Analysis.* (2021). Available online at: https://www.crd.york.ac.uk/prospero/display_record.php?ID=CRD42021244647 (accessed January 30, 2021). 10.3389/fmed.2022.899821 PMC9082645 35547228

[27] Stroup DF Berlin JA Morton SC Olkin I Williamson GD Rennie D Meta-analysis of observational studies in epidemiology: a proposal for reporting. meta-analysis of observational studies in epidemiology (MOOSE) group. *JAMA.* (2000) 283:2008–12. 10.1001/jama.283.15.2008 10789670

[28] Centers for Disease Control and Prevention. *What is Diabetes?* Atlanta, GA: Centers for Disease Control and Prevention (2020).

[29] The Ottawa Hospital Research Institute. *The Newcastle-Ottawa Scale (NOS) for Assessing the Quality of Nonrandomised Studies in Meta-Analyses.* Ottawa: The Ottawa Hospital Research Institute (2021).

[30] Zheng S Qiu M Wu JHY Pan XF Liu X Sun L Long-chain omega-3 polyunsaturated fatty acids and the risk of heart failure. *Ther Adv Chronic Dis.* (2022) 13:20406223221081616. 10.1177/20406223221081616 35321400 PMC8935400

[31] Zhu H Zheng H Xu T Liu X Liu H. Effects of statins in primary and secondary prevention for venous thromboembolism events: a meta analysis. *Vascul Pharmacol.* (2022) 142:106931. 10.1016/j.vph.2021.106931 34763100

[32] Arnedo-Pena A Juan-Cerdán JV Romeu-García MA García-Ferrer D Holguín-Gómez R Iborra-Millet J Vitamin D status and incidence of tuberculosis infection conversion in contacts of pulmonary tuberculosis patients: a prospective cohort study. *Epidemiol Infect.* (2015) 143:1731–41. 10.1017/S0950268814002386 25274036 PMC9507222

[33] Bennett RJ Brodine S Waalen J Moser K Rodwell TC. Prevalence and treatment of latent tuberculosis infection among newly arrived refugees in San Diego County, January 2010-October 2012. *Am J Public Health.* (2014) 104:e95–102. 10.2105/AJPH.2013.301637 24524534 PMC4025726

[34] Chan-Yeung M Cheung AH Dai DL Chan FH Kam KM Tam CM Prevalence and determinants of positive tuberculin reactions of residents in old age homes in Hong Kong. *Int J Tuberc Lung Dis.* (2006) 10:892–8. 16898374

[35] El-Sokkary RH Abu-Taleb AM El-Seifi OS Zidan HE Mortada EM El-Hossary D Assessing the prevalence of latent tuberculosis among health care providers in Zagazig City, Egypt Using tuberculin skin test and quantiferon-TB gold in-tube test. *Cent Eur J Public Health.* (2015) 23:324–30. 10.21101/cejph.a4101 26841146

[36] Hensel RL Kempker RR Tapia J Oladele A Blumberg HM Magee MJ. Increased risk of latent tuberculous infection among persons with pre-diabetes and diabetes mellitus. *Int J Tuberc Lung Dis.* (2016) 20:71–8. 10.5588/ijtld.15.0457 26688531 PMC5652325

[37] Khawcharoenporn T Apisarnthanarak A Phetsuksiri B Rudeeaneksin J Srisungngam S Mundy LM. Tuberculin skin test and quantiferon-TB gold in-tube test for latent tuberculosis in Thai HIV-infected adults. *Respirology.* (2015) 20:340–7. 10.1111/resp.12442 25428131

[38] Salindri AD Haw JS Amere GA Alese JT Umpierrez GE Magee MJ. Latent tuberculosis infection among patients with and without type-2 diabetes mellitus: results from a hospital case-control study in Atlanta. *BMC Res Notes.* (2021) 14:252. 10.1186/s13104-021-05662-0 34193265 PMC8247096

[39] Shu CC Wu VC Yang FJ Pan SC Lai TS Wang JY Predictors and prevalence of latent tuberculosis infection in patients receiving long-term hemodialysis and peritoneal dialysis. *PLoS One.* (2012) 7:e42592. 10.1371/journal.pone.0042592 22916137 PMC3423405

[40] Suwanpimolkul G Grinsdale JA Jarlsberg LG Higashi J Osmond DH Hopewell PC Association between diabetes mellitus and tuberculosis in United States-Born and foreign-born populations in San Francisco. *PLoS One.* (2014) 9:e114442. 10.1371/journal.pone.0114442 25478954 PMC4257695

[41] Ting WY Huang SF Lee MC Lin YY Lee YC Feng JY Gender disparities in latent tuberculosis infection in high-risk individuals: a cross-sectional study. *PLoS One.* (2014) 9:e110104. 10.1371/journal.pone.0110104 25369472 PMC4219689

[42] Wang JY Shu CC Lee CH Yu CJ Lee LN Yang PC Interferon-gamma release assay and Rifampicin therapy for household contacts of tuberculosis. *J Infect.* (2012) 64:291–8. 10.1016/j.jinf.2011.11.028 22207002

[43] Getahun H Matteelli A Abubakar I Aziz MA Baddeley A Barreira D Management of latent Mycobacterium tuberculosis infection: WHO guidelines for low tuberculosis burden countries. *Eur Respir J.* (2015) 46:1563–76. 10.1183/13993003.01245-2015 26405286 PMC4664608

[44] Cooper AM. Cell-mediated immune responses in tuberculosis. *Annu Rev Immunol.* (2009) 27:393–422. 10.1146/annurev.immunol.021908.132703 19302046 PMC4298253

[45] Kumar Nathella P Babu S. Influence of diabetes mellitus on immunity to human tuberculosis. *Immunology.* (2017) 152:13–24. 10.1111/imm.12762 28543817 PMC5543489

[46] Kumar NP George PJ Kumaran P Dolla CK Nutman TB Babu S. Diminished systemic and antigen-specific type 1, type 17, and other proinflammatory cytokines in diabetic and prediabetic individuals with latent Mycobacterium tuberculosis infection. *J Infect Dis.* (2014) 210:1670–8. 10.1093/infdis/jiu329 24907382 PMC4215076

[47] O’Garra A Redford PS McNab FW Bloom CI Wilkinson RJ Berry MP. The immune response in tuberculosis. *Annu Rev Immunol.* (2013) 31:475–527. 10.1146/annurev-immunol-032712-095939 23516984

[48] Kumar NP Moideen K George PJ Dolla C Kumaran P Babu S. Impaired cytokine but enhanced cytotoxic marker expression in mycobacterium tuberculosis-induced CD8+ T Cells in individuals with type 2 diabetes and latent mycobacterium tuberculosis infection. *J Infect Dis.* (2016) 213:866–70. 10.1093/infdis/jiv484 26486635 PMC4747616

[49] Kumar NP Moideen K George PJ Dolla C Kumaran P Babu S Coincident diabetes mellitus modulates Th1-, Th2-, and Th17-cell responses in latent tuberculosis in an IL-10- and TGF-β-dependent manner. *Eur J Immunol.* (2016) 46:390–9. 10.1002/eji.201545973 26518995 PMC6340057

[50] Grotzke JE Lewinsohn DM. Role of CD8+ T lymphocytes in control of Mycobacterium tuberculosis infection. *Microbes Infect.* (2005) 7:776–88. 10.1016/j.micinf.2005.03.001 15823514

[51] Lin PL Flynn JL. CD8 T cells and Mycobacterium tuberculosis infection. *Semin Immunopathol.* (2015) 37:239–49. 10.1007/s00281-015-0490-8 25917388 PMC4439333

[52] Kumar NP Moideen K Dhakshinraj SD Banurekha VV Nair D Dolla C Profiling leucocyte subsets in tuberculosis-diabetes co-morbidity. *Immunology.* (2015) 146:243–50. 10.1111/imm.12496 26095067 PMC4582965

[53] Orme IM. Aging and immunity to tuberculosis: increased susceptibility of old mice reflects a decreased capacity to generate mediator T lymphocytes. *J Immunol.* (1987) 138:4414–8. 3495592

[54] Baussano I Nunn P Williams B Pivetta E Bugiani M Scano F Tuberculosis among health care workers. *Emerg Infect Dis.* (2011) 17:488–94. 10.3201/eid1703.100947 21392441 PMC3298382

[55] Nasreen S Shokoohi M Malvankar-Mehta MS. Prevalence of latent tuberculosis among health care workers in high burden countries: a systematic review and meta-analysis. *PLoS One.* (2016) 11:e0164034. 10.1371/journal.pone.0164034 27711155 PMC5053544

[56] Ahmad M. *Tuberculosis (TB) Trends Among Refugee, Other Foreign-Born, and US-Born Cases in DeKalb County During 2004-2015.* Atlanta, GA: Georgia State University (2016).

[57] Zhang Z Xu X Ni H. Small studies may overestimate the effect sizes in critical care meta-analyses: a meta-epidemiological study. *Crit Care.* (2013) 17:R2. 10.1186/cc11919 23302257 PMC4056100

[58] Cheng C Tao Z Zhijian W Peng H Kong W Zhou Y The LTBI rate differences among male and female subjects among different age groups. *PLoS One.* (2015) 10:e0141511. 10.1371/journal.pone.0141511 26505997 PMC4624631

[59] Tabák AG Herder C Rathmann W Brunner EJ Kivimäki M. Prediabetes: a high-risk state for diabetes development. *Lancet.* (2012) 379:2279–90. 22683128 10.1016/S0140-6736(12)60283-9 PMC3891203

[60] Whiting DR Guariguata L Weil C Shaw J. IDF diabetes atlas: global estimates of the prevalence of diabetes for 2011 and 2030. *Diabetes Res Clin Pract.* (2011) 94:311–21. 10.1016/j.diabres.2011.10.029 22079683

[61] Kumar NP Moideen K Dolla C Kumaran P Babu S. Prediabetes is associated with the modulation of antigen-specific Th1/Tc1 and Th17/Tc17 responses in latent Mycobacterium tuberculosis infection. *PLoS One.* (2017) 12:e0178000. 10.1371/journal.pone.0178000 28558065 PMC5448753

[62] Huang Y Cai X Mai W Li M Hu Y. Association between prediabetes and risk of cardiovascular disease and all cause mortality: systematic review and meta-analysis. *BMJ.* (2016) 355:i5953. 10.1136/bmj.i5953 27881363 PMC5121106

[63] Cai X Zhang Y Li M Wu JH Mai L Li J Association between prediabetes and risk of all cause mortality and cardiovascular disease: updated meta-analysis. *BMJ.* (2020) 370:m2297. 10.1136/bmj.m2297 32669282 PMC7362233

[64] Cai X Liu X Sun L He Y Zheng S Zhang Y Prediabetes and the risk of heart failure: a meta-analysis. *Diabetes Obes Metab.* (2021) 23:1746–53. 10.1111/dom.14388 33769672

[65] Mai L Wen W Qiu M Liu X Sun L Zheng H Association between prediabetes and adverse outcomes in heart failure. *Diabetes Obes Metab.* (2021) 23:2476–83. 10.1111/dom.14490 34227220

[66] Beagley J Guariguata L Weil C Motala AA. Global estimates of undiagnosed diabetes in adults. *Diabetes Res Clin Pract.* (2014) 103:150–60. 10.1016/j.diabres.2013.11.001 24300018

