# Supplementary material for: The Association Between Diabetes Mellitus and the Risk of Latent Tuberculosis Infection: A Systematic Review and Meta-Analysis
Source: Front Med (Lausanne). 2022 Apr 25;9:899821. doi: 10.3389/fmed.2022.899821 (PMC9082645; doi:10.3389/fmed.2022.899821)
Supplement: Supplementary file 1 [file Table_1.DOCX]

**Supplementary Table 1. Search strategies and search results for each database**

| **Database** | **Search Strategies** | **Search results** |
| --- | --- | --- |
| PubMed | (((Diabetes Mellitus[Title/Abstract]) OR (diabetes[Title/Abstract])) AND ((((((Tuberculin Test[Title/Abstract]) OR (Interferon-gamma Release Tests[Title/Abstract])) OR (Latent Tuberculosis[Title/Abstract])) OR (tuberculin skin test[Title/Abstract])) OR (interferon gamma release assay[Title/Abstract]))) OR (Latent Tuberculosis[Title/Abstract]) | 4324 studies |
| Embase | ('tuberculin test':ab,ti OR 'interferon-gamma release tests':ab,ti OR 'latent tuberculosis':ab,ti OR 'tuberculin skin test':ab,ti OR 'interferon gamma release assay':ab,ti) AND ('diabetes mellitus':ab,ti OR 'diabetes':ab,ti) OR 'latent tuberculosis':ab,ti | 5907 studies |
| Cochrane Library | #1 (tuberculin test):ti,ab,kw OR (interferon-gamma release tests):ti,ab,kw OR (latent tuberculosis):ti,ab,kw OR (tuberculin skin test):ti,ab,kw OR (interferon gamma release assay):ti,ab,kw  #2 (diabetes mellitus):ti,ab,kw OR (diabetes):ti,ab,kw  #3 (latent tuberculosis):ti,ab,kw  Search (#1 and #2) or #3 | 500 studies |
| Web of Science | ((AB=“Tuberculin Test” OR AB=“Interferon-gamma Release Tests” OR AB=“Latent Tuberculosis” OR AB=“tuberculin skin test” OR AB=“interferon gamma release assay”) AND (AB=“Diabetes Mellitus” OR AB=“diabetes”)) OR (AB=“Latent Tuberculosis”) | 4206 studies |
