# Supplementary material for: The Association Between Diabetes Mellitus and the Risk of Latent Tuberculosis Infection: A Systematic Review and Meta-Analysis
Source: Front Med (Lausanne). 2022 Apr 25;9:899821. doi: 10.3389/fmed.2022.899821 (PMC9082645; doi:10.3389/fmed.2022.899821)
Supplement: Supplementary file 2 [file Table_2.DOCX]

**Supplementary Table 2. Crude and Adjusted Odds Ratios From included Studies**

| **Study** | **Crude Odds Ratio (95% Confidence Interval)** | **Adjusted Odds Ratio (95% Confidence Interval)** | **Adjusted Variables** |
| --- | --- | --- | --- |
| Arnedo-Pena | 2.85 (0.38-21.44) | .. | .. |
| Argita D. Salindri | 0.59 (0.19-2.04) | 0.45 (0.13-1.71) | age and sex |
| Barron | 2.70 (1.76-4.14) | 1.90 (1.15-3.14) | age, sex, smoking status, history of active TB, and foreign-born status |
| Bennett | 3.32 (2.44-4.52) | 1.58 (1.13-2.20) | Birth region, age, gender, education, malignancy, HIV, end-stage renal disease, smoking |
| Chan-Yeung | 1.38 (1.18-1.61) | 1.15 (0.97-1.37) | Age, marital status, education, place of birth, smoking, drug abuse, past tuberculosis, cardiovascular disease, arthritis, fracture, ischemic heart disease, COPD, cancer, liver disease, BMI, feeding method, Norton score |
| El-Sokkary | .. | .. | .. |
| Hensel RL | 2.19 (1.22-3.94) | 2.27 (1.15-4.48) | age, sex, BMI, TB incidence in country of origin, smoking status, and vitamin D level. |
| Jackson | .. | 1.15 (1.03-1.29) | age, sex, ethnicity, body mass index and the presence of other immunocompromising conditions |
| Khawcharoenporn | 1.62 (0.28-9.22) | 1.82 (0.32-10.53) | Sex, smoking |
| Kubiak | 1.24 (1.04-1.48) | 1.20 (0.99-1.45) | age, sex, body mass index category, smoking, and hazardous alcohol use |
| Lin | 2.68 (1.94-3.71) | 1.67 (1.18-2.38) | age, gender, smoking status, chronic kidney disease, history of TB and TB contact |
| Martinez | 2.0 (1.5-2.6) | 1.5 (1.0-2.2) | age, gender, household contact with a tuberculosis case, birthplace (United States or foreign born), smoking status, family size |
| Nanth | .. | 1.88 (1.25–2.82) | gender, age, ethnicity, smoking status and BMI |
| Shivakumar | .. | .. | .. |
| Shu, C. C. | .. | .. | .. |
| Stockbridge | 1.15 (1.10-1.20)* | .. | .. |
| Suwanpimolkul | .. | .. | .. |
| Ting, W. Y | .. | .. | .. |
| Wang, J. Y. | .. | .. | .. |
| Yeon | 2.837 (1.001–8.044) | .. | .. |

*99.9% Confidence Interval. TB, tuberculosis; HIV, human immunodeficiency virus; COPD, chronic obstructive pulmonary disease; BMI, Body Mass Index.
