# Supplementary material for: The Association Between Diabetes Mellitus and the Risk of Latent Tuberculosis Infection: A Systematic Review and Meta-Analysis
Source: Front Med (Lausanne). 2022 Apr 25;9:899821. doi: 10.3389/fmed.2022.899821 (PMC9082645; doi:10.3389/fmed.2022.899821)
Supplement: Supplementary file 3 [file Table_3.DOCX]

**Supplementary Table 3**. Risk of bias of studies involved using the Newcastle-Ottawa quality assessment scale

| Cross-sectional studies | | | | | | | | | | | | | | | | | | | | |
| --- | --- | --- | --- | --- | --- | --- | --- | --- | --- | --- | --- | --- | --- | --- | --- | --- | --- | --- | --- | --- |
| first author | 1) | 2) | | 3) | | 4) | | 5) | | 6) | 7) | 8) | | 9) | | 10) | | 11) | scores | risk of bias |
| Salindri | yes | yes | | yes | | no | | no | | yes | yes | yes | | no | | yes | | no | 7 | moderate |
| Barron | yes | yes | | yes | | yes | | yes | | yes | yes | yes | | yes | | yes | | no | 10 | low |
| Bennett | yes | yes | | yes | | yes | | no | | yes | yes | yes | | no | | yes | | no | 8 | low |
| Chan-Yeung | yes | no | | no | | yes | | yes | | no | no | yes | | no | | yes | | no | 5 | moderate |
| El-Sokkary | yes | yes | | yes | | yes | | no | | yes | yes | no | | no | | yes | | no | 7 | moderate |
| Hensel | yes | yes | | yes | | yes | | no | | yes | no | yes | | yes | | yes | | no | 8 | low |
| Jackson | yes | yes | | yes | | yes | | yes | | no | yes | yes | | no | | yes | | no | 8 | low |
| Kubiak | yes | yes | | yes | | no | | yes | | yes | yes | yes | | no | | yes | | yes | 9 | low |
| Lin | yes | yes | | yes | | no | | yes | | yes | yes | yes | | no | | yes | | no | 8 | low |
| Martinez | yes | yes | | yes | | no | | yes | | yes | yes | yes | | no | | yes | | no | 8 | low |
| Nanth | yes | yes | | yes | | yes | | yes | | yes | yes | yes | | no | | yes | | no | 9 | low |
| Shivakumar | yes | yes | | yes | | yes | | no | | yes | yes | yes | | no | | yes | | no | 8 | low |
| Shu | yes | yes | | yes | | no | | no | | yes | yes | no | | no | | yes | | no | 6 | moderate |
| Stockbridge | yes | yes | | yes | | yes | | yes | | yes | yes | no | | no | | yes | | no | 8 | low |
| Suwanpimolkul | yes | yes | | yes | | yes | | yes | | yes | no | yes | | no | | yes | | no | 8 | low |
| Ting | yes | yes | | yes | | yes | | no | | yes | yes | yes | | no | | yes | | no | 8 | low |
| Yeon | yes | no | | yes | | no | | yes | | yes | yes | yes | | no | | yes | | no | 7 | moderate |
| Cohort studies | | | | | | | | | | | | | | | | | | | | |
| first author | Selection | | | | | | | | Comparability | | | | Outcome | | | | | | scores | risk of bias |
|  | 1) | | 2) | | 3) | | 4) | | 5) | | | | 6) | | 7) | | 8) | |  |  |
| Arnedo-Pena | 1 | | 1 | | 1 | | 1 | | 1 | | | | 1 | | 1 | | 1 | | 8 | Low |
| Khawcharoenporn | 0 | | 1 | | 1 | | 1 | | 1 | | | | 1 | | 1 | | 1 | | 7 | Low |
| Wang | 0 | | 1 | | 1 | | 1 | | 2 | | | | 1 | | 1 | | 0 | | 7 | Low |

For cross-sectional studies:

1) Define the source of information (survey, record review)

2) List inclusion and exclusion criteria for exposed and unexposed subjects (cases and controls) or refer to previous publications

3) Indicate time period used for identifying patients

4) Indicate whether or not subjects were consecutive if not population-based

5) Indicate if evaluators of subjective components of study were masked to other aspects of the status of the participants

6) Describe any assessments undertaken for quality assurance purposes (e.g., test/retest of primary outcome measurements)

7) Explain any patient exclusions from analysis

8) Describe how confounding was assessed and/or controlled.

9) If applicable, explain how missing data were handled in the analysis

10) Summarize patient response rates and completeness of data collection

11) Clarify what follow-up, if any, was expected and the percentage of patients for which incomplete data or follow-up was obtained

For cohort studies:

1) Representativeness of the exposed cohort

*a) truly representative of the average _______________ (describe) in the community

*b) somewhat representative of the average ______________ in the community

c) selected group of users eg nurses, volunteers

d) no description of the derivation of the cohort

2) Selection of the non exposed cohort

*a) drawn from the same community as the exposed cohort

b) drawn from a different source

c) no description of the derivation of the non exposed cohort

3) Ascertainment of exposure

*a) secure record (eg surgical records)

*b) structured interview

c) written self report

d) no description

4) Demonstration that outcome of interest was not present at start of study

*a) yes

b) no

5) Comparability of cohorts on the basis of the design or analysis

*a) study controls for the most important factor)

*b) study controls for any additional factor (This criteria could be modified to indicate specific control for a second important factor.)

6) Assessment of outcome

*a) independent blind assessment

*b) record linkage

c) self report

d) no description

7) Was follow-up long enough for outcomes to occur

*a) yes (select an adequate follow up period for outcome of interest)

b) no

8) Adequacy of follow up of cohorts

*a) complete follow up - all subjects accounted for

*b) subjects lost to follow up unlikely to introduce bias - small number lost - > 70 % follow up, or description provided of those lost)

c) follow up rate < 70% and no description of those lost

d) no statement
